# Resident perspectives on duty hour limits and attributes of their learning environment

**DOI:** 10.1186/1472-6920-14-S1-S7

**Published:** 2014-12-11

**Authors:** Ingrid Philibert

**Affiliations:** 1 Department of Field Activities, Accreditation Council for Graduate Medical Education, 515 North State Street, Ste. 2000, Chicago, IL 60654, USA

## Abstract

**Background:**

Residents are stakeholders in the debate surrounding duty hour restrictions, yet few studies have assessed their perspective on their programs’ efforts to comply with them.

**Objectives:**

This paper explores learners’ perceptions of the attributes of their programs in relation to duty hour compliance, and looks for evidence whether residents view duty hour limits as important to patient safety.

**Methods:**

A grounded-theory framework was used to analyze learners’ comments about programs’ compliance with US duty hour limits. Data were collected by ACGME in 2011, using resident consensus lists of program strengths and opportunities for improvement generated prior to accreditation site visits. The data set for this analysis encompasses 112 core and 69 subspecialty programs where these lists mentioned duty hours.

**Results:**

The analysis compared programs where residents viewed duty hour compliance as a strength, and programs where it was identified as an opportunity for improvement. Programs in the first group were characterized by clinical efficiency, responsiveness to problems, and a collegial environment that contributed to residents’ ability to meet clinical and learning goals within the restrictions. These attributes were lacking in the second group, and residents also commented on onerous duty hour reporting. Learners did not associate duty hour compliance with patient safety, and the few comments in this area centred almost exclusively on the presence or absence of supervision when junior residents first assumed clinical duties.

**Conclusion:**

The findings have practical implications for programs that wish to enhance their learning and patient care environment, and suggest areas for future research.

## Background

Postgraduate medical education prepares physicians for practice in a clinical specialty [1]. In this phase of training, young physicians – often for the first time – are exposed to the demands of real-life practice, including long work hours [2,3]. In 1999, the Institute of Medicine (IOM) drew public attention to the issue of patient safety with the release of a report that implicated medical errors and adverse events as a cause or contributing factor in 44,000 to 98,000 deaths annually in the United States; the total associated costs of these events was estimated at US$17 to US$29 billion [4]. Although the IOM report did not implicate resident physicians or their long work hours, it generated debate in the health care community and the media about the safety and quality of care in US hospitals. In 2001, legislation to regulate resident hours was introduced in both houses of the US Congress [5,6]. In response, in 2003 the Accreditation Council for Graduate Medical Education (ACGME), which accredits postgraduate medical education in the United States, instituted common duty hour limits for all accredited specialties, including a weekly limit of 80 hours, one day off in seven, and in-house call no more frequently than every third night [7].

In 2008, an IOM report on resident duty hours again highlighted long work hours and fatigue as factors that contributed to the incidence of medical errors [8]. In response, the ACGME developed stricter limits on continuous duty hours, including a 16-hour limit for first-year residents, along with enhancements in supervision, alertness management, and transitions of care as elements of a comprehensive approach to increase patient safety in teaching settings and to promote resident learning and professional development [9]. The new standards became effective in July 2011.

Residents’ perspectives on work hours and their learning environment have been explored in various studies, the results of which have both provided support for the limits [10,11] and raised concerns that they may compromise the attainment of safety goals [12,13] and impede resident learning [14-16]. Most studies to date have used questionnaires, limiting residents’ responses to predetermined categories. An exception is the work by Yedidia and colleagues, who assessed residents’ perceptions of duty hour regulations in New York State in the early 1990s [17]. The residents’ responses revealed mixed feelings about the duty hour limits; including “concerns about leaving patients at critical junctures in their care, regard for the workload of colleagues, and uneasiness about the educational consequences” [17]. Testimony to the ACGME Duty Hours Task Force nearly 20 years later again highlighted conflict between, on the one hand, compliance with the limits and, on the other hand, issues of professional commitment to patients and educational concerns that caused residents to remain beyond the limits (Oral testimony to the ACGME Task Force On Duty Hours and the Learning Environment, Chicago (IL), June 9–10, 2009; June 9–10; unpublished).

This article presents an analysis of aggregated comments related to duty hours and the working and learning environment collected by the ACGME in the six months before the implementation of the 2011 duty hour standards. It explores residents’ perceptions of the limits in the context of other program attributes as stated in their own words. Concerns about work hours and patient safety permeated the 2008 IOM report, and this study also looks for evidence as to whether residents view duty hour limits as important to patient safety.

## Methods

Data were collected between January and June 2011, as part of a pilot study to enhance resident input into ACGME site visits. The ACGME asked residents in programs undergoing a site visit to develop a consensus list of up to five program strengths and five opportunities for improvement. The lists were shared directly (and confidentially) with the site visitor. The collection of this information has since been implemented as standard for all site visits.

To produce the data set for this study, site visitors transmitted the consensus lists received from the residents to an ACGME staff member (JE), who removed identifiers and sorted the data. De-identification ensured that the analysis was not influenced by known information about programs, such as compliance with duty hour standards or the learning environment they provide. The subset of data used in the present study encompassed all lists that mentioned duty hours, sorted into two groups: programs for which residents commented on duty hour compliance as a strength, and programs for which duty hour compliance was identified as an opportunity for improvement. The author and another member of the ACGME staff (JS) analyzed the aggregated, de-identified comments. The analysis used a grounded- theory framework [18,19], focusing on statements that related to residents’ perceptions of duty hours and attributes of their working and learning environment. Comments were coded and grouped, with the aim of identifying concepts and themes. This approach sought to promote theoretical validity (the degree to which an explanation developed fits the data and is plausible and credible) [20]. Besides the author, two ACGME staff members (JE and JS) independently reviewed the themes and assigned quotes for consistency and theoretical validity; disagreements were resolved by discussion, in keeping with accepted approaches for qualitative research [21]. A draft of the report was reviewed by two residents and two members of the ACGME field staff for plausibility of the comments and linkages. Their review resulted in several changes to the titles assigned to the grouped comments.

## Results

The overall data set encompassed 206 core and 193 subspecialty programs, and the consensus lists for 112 core specialty and 69 subspecialty (fellowship) programs (55% and 36% of the total, respectively), included comments about duty hours or the duty hour limits. The majority of specialties and many subspecialties were represented. Programs in core specialties were more likely than subspecialty programs to have a mention of duty hours among the consensus list on strengths and opportunities for improvement. Some core specialties in which residents rarely reach the duty hour limits (Pathology, Dermatology, Psychiatry) were also sparsely represented in the data set.

The presentation of the results is organized according to the attributes of residency and fellowship programs and the residents’ learning and working environment. It contrasts programs in which duty hour compliance was mentioned as a strength with programs in which duty hour compliance was viewed as an area improvement. Direct quotations are interspersed with the analysis to present residents’ perceptions in their own words.

### Program responsiveness and improvement

Programs for which residents reported duty hour compliance as a strength ensured that residents adhered with the duty hour limits and were not overworked. Residents reported that these programs achieved compliance without negatively influencing their clinical experience and sense of responsibility to their patients. These programs also emphasized accurate reporting of resident hours. Residents noted that changes had been made to rotations and other program elements to address compliance problems.

“Challenging cases; good mix of education v. service responsibilities, good learning opportunities within the duty hour limits.”

“We have made great strides in work hour restriction compliance. Residents are compelled to log their hours honestly, and logs are evaluated often in case changes need to be made. Rotations have been adjusted to allow resident compliance.”

“We see the Program leadership, our director and the department chairman, as exceptional assets. Their support of the residents, in both professional and personal issues, is robust and unfailing. Both are receptive to suggestions and accommodating to requests from the residents and actively work to address each and every concern mentioned.”

In contrast, programs for which duty hour compliance was reported as an area for improvement had expectations for strict compliance with the limits; this was often reported in combination with residents commenting on excessive time spent on duty hour reporting, inefficiency in data collection systems. Of particular concern to residents was a lack of responsiveness on the part of program leaders, expressed as an absence of interventions to address duty hour compliance problems and to, more generally, improve the educational experience.

“The requirements for documentation of cases, duty hours, etc are burdensome.”

“The residents as a whole feel as though we have no clear authoritative advocate who can help us lobby for our own interests with administration.”

### Clinical load and efficiency

In a number of programs for which residents identified duty hour compliance as a strength, residents commented on the efficiency of their clinical environment and on how they were supported in their clinical work.

“Extensive multidisciplinary teams/extender support in the clinical setting (for example, PharmD and Nurse Practitioner and discharge planner that round daily with CCU teams).”

In contrast, residents who had a negative perception of the effect of duty hour limits and their ability to learn frequently reported problems with patient flow and lack of efficiency, particularly in the clinic and operating rooms

“Patient flow and lack of efficiency, particularly in the clinic and operating rooms are a common reason residents exceed their duty hours.”

“Our clinic’s flow of patients is horrendous, with many patients commonly waiting at least 30-45 minutes before seeing their physician; it’s common for residents to be running an hour behind schedule. Some of the steps in the registration process and nursing assessment of the patient are the cause of these delays. This affects our learning and ability to practice medicine.”

Residents also commented on the time-consuming nature and inefficiency of electronic medical records, and on supervision and staffing issues in clinical settings as attributes of clinical systems that contributed to duty hour non-compliance.

“[We have n]o administrative time to deal with all the paperwork, [EMR] inbox responsibilities, and extra activities like logging duty hours, procedures, e-Learning, etc. This is usually put off until the end of clinic, which makes our days longer, or the weekends. Some of those activities can only be done through work computers and we can’t log on remotely, which means it can’t easily be done on days off.”

“We need better discharge planning/paperwork coordination. This is a predominant problem in [our specialty], leading to increase[d] work load on residents and eventually causing residents to be over their duty hours (very common).”

“A formal system for staffing inpatients during clinic hours is lacking. Any inpatient case that needs to be staffed during the day requires the resident to find a willing faculty member, which is more difficult than one would think (there is always a faculty member designated for after-hour and weekend cases).”

Inefficiencies in physical settings, particularly distance between inpatient units, and between hospitals and ambulatory settings, and the resulting commuting times, were also commonly cited as factors that increased residents’ hours without contributing to learning.

“Although the training opportunity at the State Hospital is excellent, the commute there from the university is time consuming (approximately 2 ½ hours a day) and trying.”

Fellows’ comments with respect to clinical issues can be sorted into two basic categories. Some viewed duty hour enforcement as a barrier to continuity of care and to transitioning to unsupervised practice after completion of training, while others mentioned long work hours and high clinical load as areas for improvement. It is possible that some comments resulted from departments shifting clinical work to fellows to ensure resident compliance with the impending 2011 duty hour limits. No particular specialties were overrepresented in these comments.

“Although the average number of new consults per day is approximately 5, the inpatient service can be very busy with the potential for days with 2-3 times that number. Given the complexity of the cases, these high-volume days, particularly several in succession, can be very taxing on the fellows and increase work hours significantly.”

“The average number of hours worked by the clinical fellows per week does not exceed 80, but variability in volume of the service often results in multiple long days in a row, and occasionally some particularly heavy weeks.”

### Collegiality, sense of shared responsibility, and teamwork

A frequent attribute of programs for which duty hour compliance was reported as a strength was collegiality: residents commented on a sense of shared responsibility for patients among the residents and, often, the faculty.

“Resident camaraderie is a major strength. This provides for a pleasant working environment in which we have fun together learning how to operate and care for the surgical patient. We also take time to get together after work and socialize on a weekly basis and on special occasions further adding to the experience of residency.”

“Collegiality among residents and faculty builds a comfortable, professional atmosphere. Faculty [model a] strong work ethic and continuing inquiry as well as peer support and patient compassion.”

A disproportionate number (10/24, 41.7%) of these reports came from Obstetrics/ Gynecology and Emergency Medicine residents, who collectively accounted for 14% of the programs in the data set. Comments from these residents also emphasized a team approach to patient care. This was the only area for which the data suggested an association between clinical specialty and residents’ observations about program strengths and opportunities for improvement. In contrast, when residents mentioned duty hour compliance as needing improvement in their program, they commented on a lack of camaraderie among the residents or between residents and faculty, and noted that this had a more general negative impact on the clinical and learning environment. Some comments again related to the geographic separation of residents on different patient care units, along with physical plant and infrastructure barriers; residents reported that these barriers had a negative effect on achieving collegiality and close interpersonal relationships among the residents and between residents and faculty.

“The faculty can be critical of their colleagues’ clinical decisions, which has makes the fellows feel uncomfortable working "between" attendings with different management strategies.”

“Geographical separation of hospitals, conferences, and administration and house staff make a sense of team and togetherness difficult to promote.”

## Discussion

This study assessed residents’ and fellows’ perceptions of duty hour limits and other attributes of their learning environment. It showed that programs for which duty hour compliance was cited as a strength had well-developed clinical infrastructure that made it possible for residents to complete their educational and clinical obligations within the allowed hours, and in many cases had made changes to improve duty hour compliance. For programs in which duty hours were mentioned as an area for improvement, comments often related to inefficiency in the clinical setting, lack of the program’s responsiveness, or the onerous nature of reporting the number of hours worked. This suggests that two sets of conditions may contribute to residents’ frustration with duty hour compliance. The first encompasses programs that make duty hour reporting detailed and onerous, but fail to address problems identified through these reports, causing residents to perceive duty hour data collection as both time-consuming and “ineffective.” The second pertains to programs that have strict expectations for duty hour compliance, but have not invested in infrastructure and support staff to address resident clinical load or efficiency. Residents in these programs perceived duty hour data collection as “dishonest,” given the low value programs appeared to place on their time. Comment on the positive aspects of programs went beyond duty hour compliance and centred on the extent to which programs provided a “real” learning environment. Faculty interest in teaching and mentoring was critically important to these positive perceptions, as was a collegial relationship among the residents and between residents and faculty.

“Our faculty is dedicated to teaching the residents. Experienced faculty [take] the extra time to teach the critical thinking skills that are so important in medicine today. Even when seeing the routine patients, effort is made to find teaching moments. There is always support when making decisions that are outside your comfort zone. We appreciate that we have not been made to feel like second rate doctors or work horses, but have been respected as peers and given appropriate guidance and feedback to ensure growth.”

Residents’ comments did not associate duty hour compliance and safety and quality of care. Residents neither commented on the patient safety benefits in the context of duty hour compliance as a strength, nor did they equate excess duty hours with patient safety concerns. Reported concerns about safety almost exclusively centred on a lack of adequate supervision by faculty or senior residents when junior residents assumed clinical duties. Residents’ comments also did not, for the most part, echo the concerns about the threats of diminished clinical competence and professionalism of graduates found in studies of perceptions of the ACGME standards [11-13,16,22,23]. Worry about preparedness for independent practice was reported to a limited extent in concerns residents voiced about the pending implementation of the ACGME’s 2011 common standards.

A strength of this study is that the statements from each individual program represents the consensus of its residents, and that the overall sample thus aggregates the opinions of a sizable sample of residents. Another strength is that the approach allowed respondents to describe their perceptions in their own words. This contrasts with the majority of studies that have used survey questions or “focused on isolated outcomes of changes in duty hours” [24].

This study has several limitations: (1) the “consensus” approach would have eliminated dissenting opinions; (2) residents volunteered the comments on duty hours, resulting in the potential for sampling bias; (3) not all programs with duty hours as a strength or opportunity for improvement in the larger sample may have been represented in sample analyzed; and (4) resident responses provided in the context of an accreditation visit may have been influenced by social desirability bias or may simply have been dishonest to mask duty hour compliance problems. This last concern is heightened by studies showing that some percentage of residents are not truthful in recording and reporting their work hours [25,26] and that in the context of an accreditation site visit residents would want their programs to be considered favourably. At the same time, the candid reporting on barriers to duty hour compliance in a sizable number of programs suggests some degree of frankness in reporting by the residents.

## Conclusions

The findings presented here have practical implications for programs wishing to enhance their learning and patient care environment. Residents in settings they perceived as ineffective in administering the duty hour standards viewed excessively rigorous compliance efforts as barrier to their professional development for independent practice, and as an ongoing irritant due to the time and energy spent on reporting, particularly when that data did not result in change in the local learning environment.

This suggests a need to focus more on clinical support and on the attributes of the environment that surrounds residents, and to seek to reduce the pressures residents face in completing clinical tasks within a restricted number of continuous hours. It appears that some specialties, particularly Obstetrics/Gynecology, with its focus on team responsibility for patients, and Emergency Medicine, with its long familiarity with shift-based approaches, could offer models for adoption or adaptation by other specialties.

These findings suggest areas for future study, including research into ways to reduce the onus on residents to report their duty hours, to more effectively incorporate electronic medical records into resident practice, and to begin the daunting task of reducing clinical inefficiencies as a common source of frustration for residents (and likely their faculty). Lack of effectiveness in the operation of clinical services, attributes of physical plants, and rotations that produce geographic separation present formidable challenges, and ask for novel approaches that place solutions within the realm of what a residency program can accomplish. Perhaps the most important areas for future research entails work to improve team coordination and team-based care in the learning environment and work to enhance supervision, which residents saw as critical to patient safety. These dimensions of programs are equally important to efforts to improve residents’ learning environment and to a national effort to ensure the safety and effectiveness of care in settings where residents learn and provide care.

## Competing interests

The author is a salaried staff member of the Accreditation Council for Graduate Medical Education; she declares no other competing interests.

## Declarations

Resources and secretariat support for this project was provided by the Royal College.

This article has been published as part of *BMC Medical Education* Volume 14 Supplement 1, 2014: Resident duty hours across borders: an international perspective. The full contents of the supplement are available online at http://www.biomedcentral.com/bmcmededuc/supplements/14/S1. This article was submitted and peer reviewed in 2012. Final acceptance for publication as part of this supplement was in 2014.

Publication of this supplement was supported and funded by the Royal College of Physicians and Surgeons of Canada. The funding agency played no role in the design, in the collection, analysis and interpretation of data; in the writing of the manuscripts; and in the decision to submit the manuscript for publication. The articles have been through the journal's standard peer review process for supplements. The Supplement Editors declare that they have no competing interests.
